# Supplementary material for: Prolonged Inhibition of the MEK1/2-ERK Signaling Axis Primes Interleukin-1 Beta Expression through Histone 3 Lysine 9 Demethylation in Murine Macrophages
Source: Int J Mol Sci. 2023 Sep 22;24(19):14428. doi: 10.3390/ijms241914428 (PMC10572145; doi:10.3390/ijms241914428)
Supplement: Supplementary file 1 [file ijms-24-14428-s001.zip › ijms-2598326-supplementary.pdf]

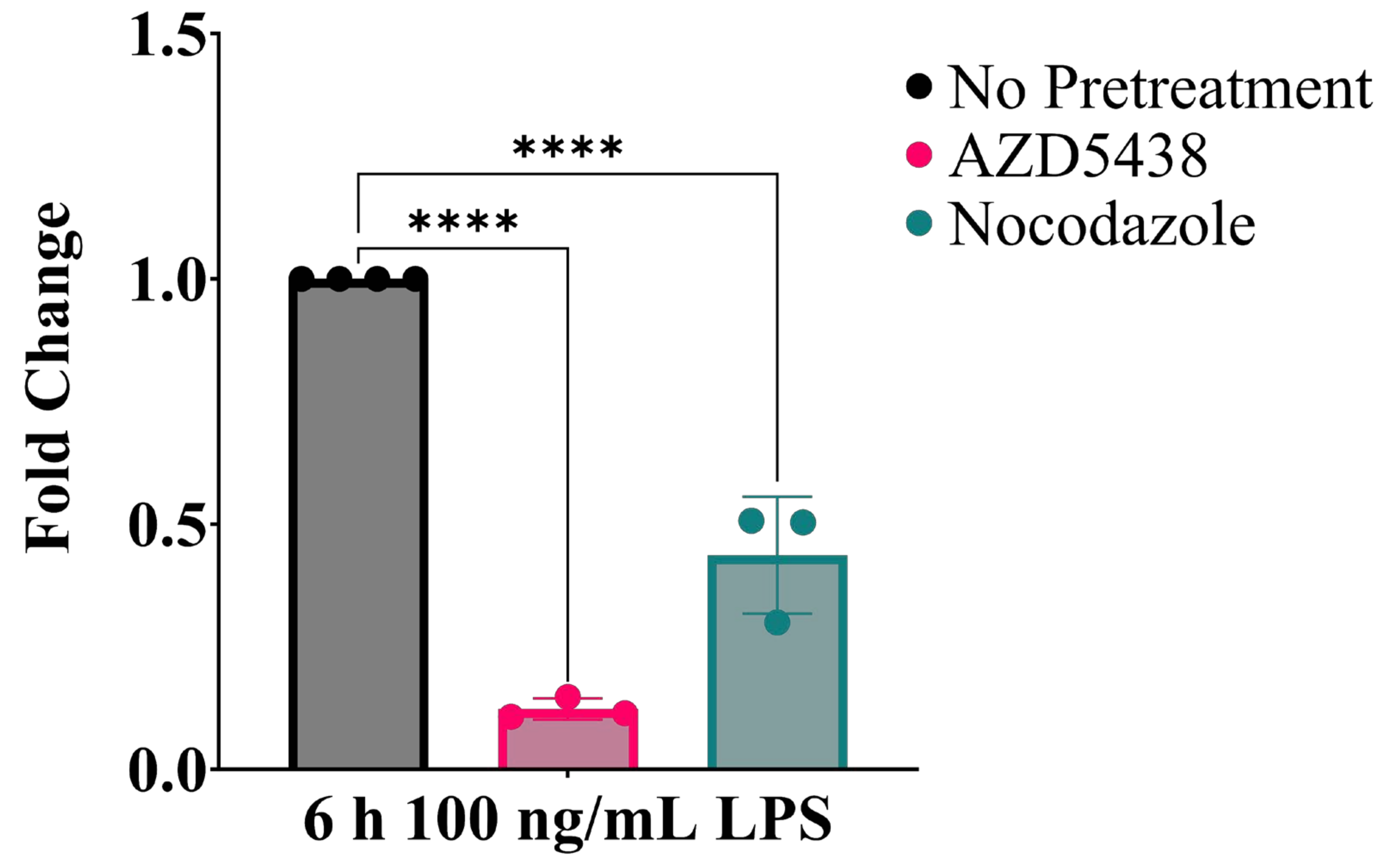

Supplemental Figure S1. Cell cycle inhibitor inhibited IL-1β mRNA expression in RAW264.7 cells. RAW264.7 cells were treated with or without the cyclin-dependent kinase inhibitor AZD5438 or the anti-mitotic agent nocodazole for 18-24 hours. Cells were washed after their treatments and then given fresh complete media containing LPS for 6 hours. I L-1β was quantified using RT-qPCR. A one-way ANOVA test with Dunnett's multiple comparisons test was done to calculate significance (*n*=3, **** p < or = 0.0001).

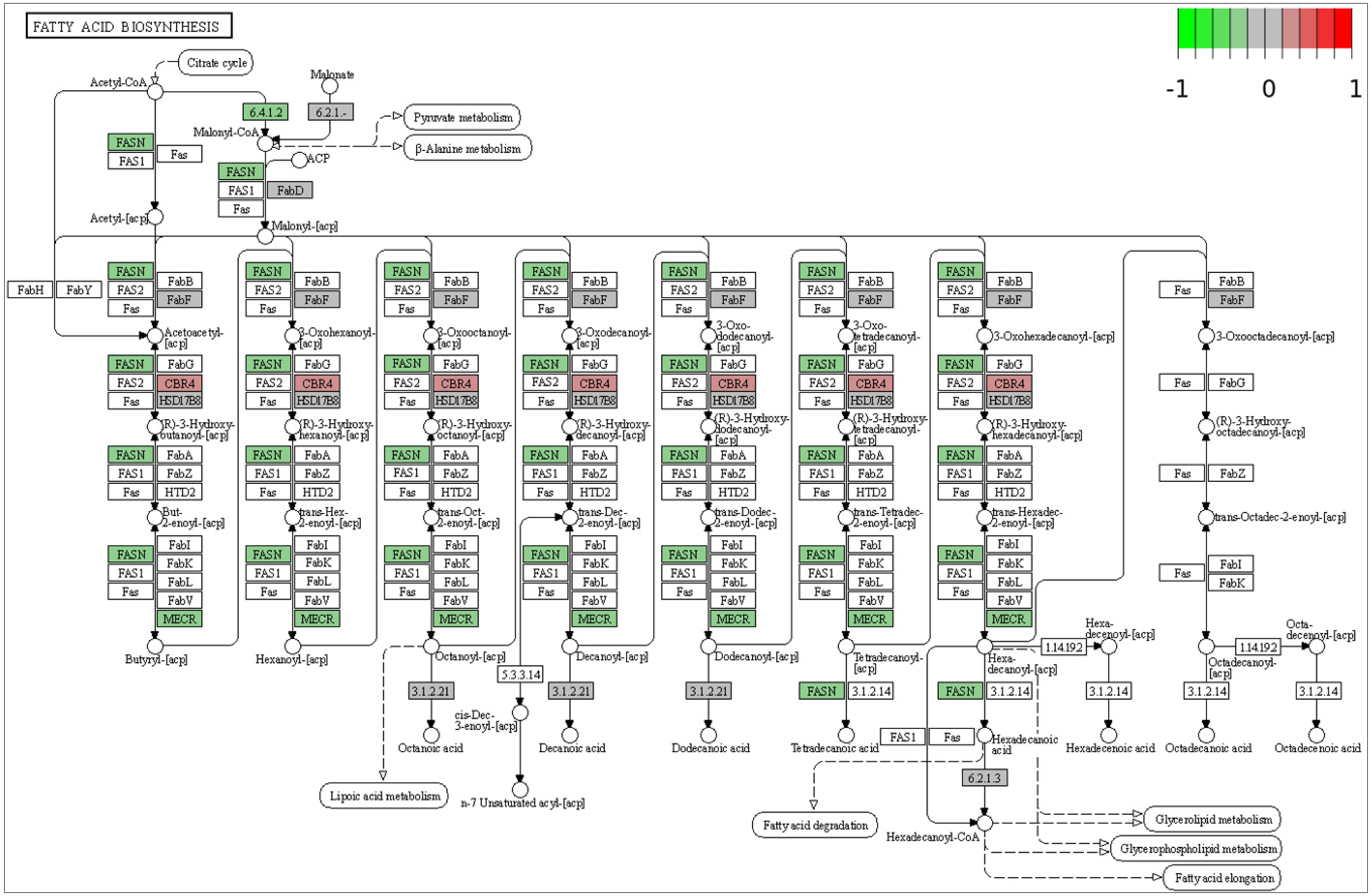

Supplemental Figure S2. Fatty acid biosynthesis pathway U0126 primed cells preferenctially downregulated genes involved in fatty acid biosynthesis. Transcriptomic analysis of RAW cells non-primed or primed by U0126 were performed as described in Reagents and Method. Differential expression of genes in fatty acid biosynthesis gene ontology was visualized by GO terms plot using the GOSEQ tool in the Galaxy platform.

Supplemental Table S1. Functional gene clusters (nodes) changed by U0126

Epigentics_heterochromatin

EnrichmentMap::Name	EnrichmentMap::NES (Dataset 1)	EnrichmentMap::fdr_qvalue (Dataset 1)
GOCC_PERICENTRIC_HETEROCHROMATIN	-1.8647	3.00E-04
GOBP_NUCLEOSOME_ASSEMBLY	-1.8636	3.00E-04
GOCC_HETEROCHROMATIN	-1.8554	4.00E-04
GOCC_SEX_CHROMOSOME	-1.8304	6.00E-04
GOCC_ESC_E_Z_COMPLEX	-1.8215	7.00E-04
GOCC_METHYLTRANSFERASE_COMPLEX	-1.7955	0.001
GOMF_HISTONE_BINDING	-1.7803	0.0013
GOCC_PRONUCLEUS	-1.7345	0.0026
GOCC_HISTONE_METHYLTRANSFERASE_COMPLEX	-1.706	0.004
GOCC_PCG_PROTEIN_COMPLEX	-1.6847	0.0054
GOMF_NUCLEOSOME_BINDING	-1.6838	0.0055
GOBP_NUCLEOSOME_ORGANIZATION	-1.6799	0.0057
GOBP NEGATIVE REGULATION OF GENE EXPRESSION	-1.6797	0.0057
GOBP_RRNA_MODIFICATION	-1.6795	0.0057
GOBP_NUCLEOLUS_ORGANIZATION	-1.6733	0.0062
GOBP_DNA_METHYLATION	-1.6689	0.0066
GOBP_HETEROCHROMATIN_ORGANIZATION	-1.6682	0.0067
GOMF_METHYLATED_HISTONE_BINDING	-1.6554	0.0078
GOCC_MLL1_2_COMPLEX	-1.6444	0.009
GOBP_CHROMATIN_REMODELING	-1.6308	0.0106
GOCC_CHROMATIN	-1.5939	0.0163
GOMF TRANSFERASE ACTIVITY TRANSFERRING ONE C	-1.5882	0.0174
GOBP_HISTONE_H3_K9_METHYLATION	-1.5882	0.0173
GOBP_CHROMATIN_ORGANIZATION	-1.5741	0.0203
GOBP_DNA_MODIFICATION	-1.5596	0.0238
GOCC_ATPASE_COMPLEX	-1.5362	0.0306
GOMF_CHROMATIN_BINDING	-1.536	0.0306
GOBP_NUCLEUS_ORGANIZATION	-1.527	0.0335
GOBP_METHYLATION	-1.519	0.0361
GOBP_HISTONE_METHYLATION	-1.5131	0.0382
GOBP EPIGENETIC REGULATION OF GENE EXPRESSIO	-1.5072	0.04
GOBP_PROTEIN_METHYLATION	-1.5022	0.0419
GOMF S ADENOSYLMETHIONINE DEPENDENT METHYL	-1.4988	0.0431

Cell cycle progression related

EnrichmentMap::Name	EnrichmentMap::NES (Dataset 1)	EnrichmentMap::fdr_qvalue (Dataset 1)
GOBP_DNA_TEMPLATED_DNA_REPLICATION	-2.3954	0
GOBP_CELL_CYCLE_DNA_REPLICATION	-2.3893	0
GOBP_MEIOTIC_CELL_CYCLE_PROCESS	-2.3462	0
GOBP_DNA_TEMPLATED_DNA_REPLICATION_MAINTENANCE_OF_FIDELITY	-2.3316	0
GOBP_NEGATIVE_REGULATION_OF_NUCLEAR_DIVISION	-2.3128	0
GOBP_MEIOSIS_I_CELL_CYCLE_PROCESS	-2.3007	0
GOBP_MEIOTIC_CELL_CYCLE	-2.2893	0
GOCC_CONDENSED_NUCLEAR_CHROMOSOME	-2.2786	0
GOBP_SPINDLE_CHECKPOINT_SIGNALING	-2.2628	0
GOCC_CONDENSED_CHROMOSOME	-2.261	0
GOBP_DNA_REPLICATION_INITIATION	-2.2566	0
GOBP_POSITIVE_REGULATION_OF_CHROMOSOME_SEGREGATION	-2.2526	0
GOBP_HOMOLOGOUS_RECOMBINATION	-2.2494	0
GOBP_REGULATION_OF_MITOTIC_SISTER_CHROMATID_SEGREGATION	-2.2345	0
GOBP_MITOTIC_CELL_CYCLE_CHECKPOINT_SIGNALING	-2.229	0
GOBP_MEIOTIC_CHROMOSOME_SEGREGATION	-2.2279	0
GOBP_SISTER_CHROMATID_SEGREGATION	-2.2257	0
GOBP_DNA_REPLICATION	-2.2188	0
GOCC_CHROMOSOME_CENTROMERIC_REGION	-2.2117	0
GOBP_REGULATION_OF_DNA_TEMPLATED_DNA_REPLICATION	-2.211	0
GOBP_NUCLEAR_CHROMOSOME_SEGREGATION	-2.2091	0
GOBP_CHROMOSOME_SEGREGATION	-2.1966	0
GOBP REGULATION OF ATTACHMENT OF SPINDLE MICROTUBULES TO KINETOCH	-2.1925	0
GOBP_CELL_CYCLE_CHECKPOINT_SIGNALING	-2.1906	0
GOBP_RECOMBINATIONAL_REPAIR	-2.1782	0
GOCC_CONDENSED_CHROMOSOME_CENTROMERIC_REGION	-2.176	0
GOCC_CHROMOSOMAL_REGION	-2.1695	0
GOMF_DNA_SECONDARY_STRUCTURE_BINDING	-2.1655	0
GOBP_SISTER_CHROMATID_COHESION	-2.1646	0
GOCC_NUCLEAR_CHROMOSOME	-2.1644	0
GOBP_NEGATIVE_REGULATION_OF_CHROMOSOME_ORGANIZATION	-2.1643	0
GOBP_CHROMOSOME_ORGANIZATION	-2.1637	0
GOMF_SINGLE_STRANDED_DNA_HELICASE_ACTIVITY	-2.1615	0
GOBP_ATTACHMENT_OF_SPINDLE_MICROTUBULES_TO_KINETOCHORE	-2.1551	0
GOBP_REGULATION_OF_CHROMOSOME_SEGREGATION	-2.1419	0
GOBP_DNA_RECOMBINATION	-2.1414	0
GOBP_REGULATION_OF_CHROMOSOME_SEPARATION	-2.1351	0
GOBP_MITOTIC_G2_DNA_DAMAGE_CHECKPOINT_SIGNALING	-2.1323	0
GOBP_CHROMOSOME_SEPARATION	-2.1249	0
GOMF_DNA_HELICASE_ACTIVITY	-2.1187	0
GOBP_CHROMOSOME_ORGANIZATION_INVOLVED_IN_MEIOTIC_CELL_CYCLE	-2.1156	0
GOBP_MITOTIC_NUCLEAR_DIVISION	-2.1131	0
GOBP_DNA_UNWINDING_INVOLVED_IN_DNA_REPLICATION	-2.1105	0
GOMF_SINGLE_STRANDED_DNA_BINDING	-2.1035	0
GOBP_PROTEIN_LOCALIZATION_TO_CHROMOSOME_CENTROMERIC_REGION	-2.0958	0
GOMF_ATP_DEPENDENT_ACTIVITY_ACTING_ON_DNA	-2.0919	0
GOBP_PROTEIN_LOCALIZATION_TO_CHROMOSOME	-2.087	0
GOBP_MITOTIC_G2_M_TRANSITION_CHECKPOINT	-2.0858	0
GOMF_DAMAGED_DNA_BINDING	-2.0782	0
GOBP_DNA_DUPLEX_UNWINDING	-2.0766	0
GOMF_CATALYTIC_ACTIVITY_ACTING_ON_DNA	-2.0757	0
GOBP_NEGATIVE_REGULATION_OF_CELL_CYCLE_PROCESS	-2.0735	0
GOBP_CELL_CYCLE_G2_M_PHASE_TRANSITION	-2.073	0
GOBP_REGULATION_OF_CELL_CYCLE_G2_M_PHASE_TRANSITION	-2.0688	0
GOBP_REGULATION_OF_NUCLEAR_DIVISION	-2.068	0
GOBP_REGULATION_OF_MITOTIC_NUCLEAR_DIVISION	-2.0679	0
GOBP_MITOTIC_METAPHASE_PLATE_CONGRESSION	-2.0617	0
GOBP_ORGANELLE_FISSION	-2.059	0
GOBP_METAPHASE_PLATE_CONGRESSION	-2.0522	0
GOMF_FOUR_WAY_JUNCTION_DNA_BINDING	-2.0479	0
GOBP_MISMATCH_REPAIR	-2.0432	0
GOBP_PROTEIN_LOCALIZATION_TO_CONDENSED_CHROMOSOME	-2.0405	0
GOBP_MITOTIC_SISTER_CHROMATID_SEPARATION	-2.0381	0
GOBP_NEGATIVE_REGULATION_OF_MITOTIC_CELL_CYCLE_PHASE_TRANSITION	-2.0213	0
GOBP_DOUBLE_STRAND_BREAK_REPAIR	-2.0176	0
GOBP_DNA_INTEGRITY_CHECKPOINT_SIGNALING	-2.0175	0
GOBP_CHROMOSOME_CONDENSATION	-2.0144	0
GOBP_DNA_REPAIR	-2.0138	0
GOBP_NEGATIVE_REGULATION_OF_MITOTIC_CELL_CYCLE	-2.0112	0
GOBP_MITOTIC_DNA_INTEGRITY_CHECKPOINT_SIGNALING	-2.0071	0
GOBP_DNA_CONFORMATION_CHANGE	-2.0038	0
GOBP_REGULATION_OF_CHROMOSOME_ORGANIZATION	-2.0011	0
GOBP_DNA_STRAND_ELONGATION	-1.999	0
GOBP_NEGATIVE_REGULATION_OF_CELL_CYCLE	-1.9923	0
GOBP_CHROMOSOME_LOCALIZATION	-1.9914	0
GOBP_POSITIVE_REGULATION_OF_CHROMOSOME_SEPARATION	-1.9913	0
GOBP_POSITIVE_REGULATION_OF_CELL_CYCLE_G2_M_PHASE_TRANSITION	-1.9848	0
GOBP_REGULATION_OF_CELL_CYCLE_CHECKPOINT	-1.9841	0
GOBP_HOMOLOGOUS_CHROMOSOME_SEGREGATION	-1.9617	1.00E-04
GOMF_DNA_REPLICATION_ORIGIN_BINDING	-1.9604	1.00E-04
GOCC_SITE_OF_DOUBLE_STRAND_BREAK	-1.9465	1.00E-04
GOBP_MITOTIC_SISTER_CHROMATID_COHESION	-1.946	1.00E-04
GOBP_REGULATION_OF_DNA_REPLICATION	-1.9433	1.00E-04
GOBP_POSITIVE_REGULATION_OF_CELL_CYCLE_PROCESS	-1.9429	1.00E-04
GOCC_SYNAPTONEMAL_STRUCTURE	-1.9422	1.00E-04
GOBP_BASE_EXCISION_REPAIR	-1.9336	1.00E-04
GOCC_SITE_OF_DNA_DAMAGE	-1.9307	1.00E-04
GOBP_MITOTIC_RECOMBINATION	-1.9306	1.00E-04
GOBP_TELOMERE_ORGANIZATION	-1.9187	1.00E-04
GOMF_ENDODEOXYRIBONUCLEASE_ACTIVITY	-1.9179	1.00E-04
GOMF_DEOXYRIBONUCLEASE_ACTIVITY	-1.9154	1.00E-04
GOBP_INTERSTRAND_CROSS_LINK_REPAIR	-1.9055	1.00E-04
GOBP_REGULATION_OF_DNA_RECOMBINATION	-1.903	2.00E-04
GOBP_NEGATIVE_REGULATION_OF_CELL_CYCLE_G2_M_PHASE_TRANSITION	-1.9022	2.00E-04
GOBP_POSTREPLICATION_REPAIR	-1.9	2.00E-04
GOCC_CHROMOSOME_TELOMERIC_REGION	-1.898	2.00E-04
GOBP_SIGNAL_TRANSDUCTION_IN_RESPONSE_TO_DNA_DAMAGE	-1.897	2.00E-04
GOBP_MITOTIC_CELL_CYCLE_PHASE_TRANSITION	-1.8955	2.00E-04
GOCC_DNA_POLYMERASE_COMPLEX	-1.8936	2.00E-04
GOBP_CELL_CYCLE_PHASE_TRANSITION	-1.8922	2.00E-04
GOBP_HOMOLOGOUS_CHROMOSOME_PAIRING_AT_MEIOSIS	-1.8886	2.00E-04
GOBP_MITOTIC_CHROMOSOME_CONDENSATION	-1.8855	2.00E-04
GOBP_REGULATION_OF_CELL_CYCLE_PHASE_TRANSITION	-1.8842	2.00E-04
GOBP_MEIOTIC_CHROMOSOME_SEPARATION	-1.8808	2.00E-04
GOBP REGULATION OF DOUBLE STRAND BREAK REPAIR VIA HOMOLOGOUS REC	-1.8806	2.00E-04
GOBP_POSITIVE_REGULATION_OF_CELL_CYCLE	-1.8776	2.00E-04
GOBP_REGULATION_OF_MITOTIC_CELL_CYCLE_PHASE_TRANSITION	-1.8759	3.00E-04
GOBP_DNA_SYNTHESIS_INVOLVED_IN_DNA_REPAIR	-1.8693	3.00E-04
GOBP_POSITIVE_REGULATION_OF_CELL_CYCLE_PHASE_TRANSITION	-1.8685	3.00E-04
GOMF_3_5_DNA_HELICASE_ACTIVITY	-1.8591	3.00E-04
GOBP_POSITIVE_REGULATION_OF_DNA_REPLICATION	-1.8587	3.00E-04
GOBP_ATTACHMENT_OF_MITOTIC_SPINDLE_MICROTUBULES_TO_KINETOCHORE	-1.8551	4.00E-04
GOBP_ESTABLISHMENT_OF_PROTEIN_LOCALIZATION_TO_CHROMOSOME	-1.8532	4.00E-04
GOBP_REGULATION_OF_MITOTIC_CELL_CYCLE	-1.8439	4.00E-04
GOMF_HELICASE_ACTIVITY	-1.8435	5.00E-04
GOBP_DNA_BIOSYNTHETIC_PROCESS	-1.8359	5.00E-04
GOBP_POSITIVE_REGULATION_OF_DNA_RECOMBINATION	-1.8258	6.00E-04
GOCC_DNA_REPAIR_COMPLEX	-1.8219	7.00E-04
GOBP_POSITIVE_REGULATION_OF_CELL_CYCLE_CHECKPOINT	-1.8213	7.00E-04
GOBP_REGULATION_OF_SPINDLE_CHECKPOINT	-1.8208	7.00E-04
GOBP_POSITIVE_REGULATION_OF_MITOTIC_CELL_CYCLE_PHASE_TRANSITION	-1.8097	8.00E-04
GOMF_DNA_POLYMERASE_BINDING	-1.8093	8.00E-04
GOBP_DETERMINATION_OF_ADULT_LIFESPAN	-1.808	8.00E-04
GOBP_REGULATION_OF_MEIOTIC_CELL_CYCLE	-1.8055	8.00E-04
GOBP_CELLULAR_RESPONSE_TO_IONIZING_RADIATION	-1.8015	9.00E-04
GOBP_ESTABLISHMENT_OF_PROTEIN_LOCALIZATION_TO_TELOMERE	-1.7905	0.0011
GOBP_REGULATION_OF_CYCLIN_DEPENDENT_PROTEIN_KINASE_ACTIVITY	-1.7856	0.0012
GOBP_RESPONSE_TO_X_RAY	-1.7848	0.0012
GOCC_TELOMERASE_HOLOENZYME_COMPLEX	-1.7817	0.0013
GOBP_REGULATION_OF_DNA_DAMAGE_CHECKPOINT	-1.7794	0.0013
GOBP_POSITIVE_REGULATION_OF_CHROMOSOME_ORGANIZATION	-1.7778	0.0013
GOBP_MALE_MEIOTIC_NUCLEAR_DIVISION	-1.7764	0.0014
GOBP_NEGATIVE_REGULATION_OF_DNA_RECOMBINATION	-1.75	0.0021
GOBP_TRANSLESION_SYNTHESIS	-1.7462	0.0023
GOBP_POSITIVE_REGULATION_OF_DNA_METABOLIC_PROCESS	-1.7441	0.0023
GOMF ENDONUCLEASE ACTIVITY ACTIVE WITH EITHER RIBO OR DEOXYRIBONUCI	-1.7404	0.0024
GOBP_DOUBLE_STRAND_BREAK_REPAIR_VIA_NONHOMOLOGOUS_END_JOINING	-1.7403	0.0024
GOBP_RESPONSE_TO_IONIZING_RADIATION	-1.7387	0.0025
GOBP_NEGATIVE_REGULATION_OF_DNA_REPAIR	-1.7387	0.0025
GOMF_CATALYTIC_ACTIVITY_ACTING_ON_A_NUCLEIC_ACID	-1.7367	0.0025
GOBP_POSITIVE_REGULATION_OF_MITOTIC_CELL_CYCLE	-1.7326	0.0027
GOBP_NEGATIVE_REGULATION_OF_DNA_METABOLIC_PROCESS	-1.7301	0.0028
GOBP_TELOMERE_MAINTENANCE_VIA_TELOMERE_LENGTHENING	-1.728	0.0029
GOBP_PROTEIN_LOCALIZATION_TO_CHROMATIN	-1.7276	0.0029
GOBP_RNA_TEMPLATED_DNA_BIOSYNTHETIC_PROCESS	-1.726	0.003
GOBP_REGULATION_OF_DNA_METABOLIC_PROCESS	-1.7223	0.0031
GOBP_POSITIVE_REGULATION_OF_DNA_BIOSYNTHETIC_PROCESS	-1.7143	0.0035
GOMF_DNA_POLYMERASE_ACTIVITY	-1.7116	0.0036
GOMF_TELOMERASE_RNA_BINDING	-1.6921	0.0049
GOBP_REGULATION_OF_DNA_REPAIR	-1.6839	0.0055
GOBP_REGULATION_OF_DOUBLE_STRAND_BREAK_REPAIR	-1.6821	0.0056
GOMF_CATALYTIC_ACTIVITY_ACTING_ON_A_TRNA	-1.6793	0.0057
GOBP_REGULATION_OF_TELOMERE_CAPPING	-1.6783	0.0058
GOBP_REGULATION_OF_RESPONSE_TO_DNA_DAMAGE_STIMULUS	-1.6724	0.0063
GOBP_RNA_5_END_PROCESSING	-1.6692	0.0066
GOBP_REGULATION_OF_TELOMERE_MAINTENANCE	-1.6655	0.0069
GOBP_TELOMERE_MAINTENANCE_VIA_RECOMBINATION	-1.6586	0.0075
GOBP_CELLULAR_RESPONSE_TO_RADIATION	-1.6572	0.0076
GOBP_TRNA_METABOLIC_PROCESS	-1.6489	0.0085
GOMF_CATALYTIC_ACTIVITY_ACTING_ON_RNA	-1.648	0.0086
GOBP_REGULATION_OF_DNA_BIOSYNTHETIC_PROCESS	-1.6457	0.0088
GOBP_NEGATIVE_REGULATION_OF_RESPONSE_TO_DNA_DAMAGE_STIMULUS	-1.6437	0.0091
GOBP POSITIVE REGULATION OF DOUBLE STRAND BREAK REPAIR VIA HOMOLOG	-1.6344	0.0102
GOBP_POSITIVE_REGULATION_OF_TELOMERE_MAINTENANCE	-1.6337	0.0103
GOMF_ATP_DEPENDENT_ACTIVITY_ACTING_ON_RNA	-1.6228	0.0117
GOMF_ENDONUCLEASE_ACTIVITY	-1.6178	0.0123
GOMF_DNA_DIRECTED_DNA_POLYMERASE_ACTIVITY	-1.6175	0.0124
GOBP_TRNA_PROCESSING	-1.6097	0.0135
GOBP_POSITIVE_REGULATION_OF_DNA_REPAIR	-1.6038	0.0146
GOBP_REGULATION_OF_TELOMERE_MAINTENANCE_VIA_TELOMERE_LENGTHENING	-1.5978	0.0157
GOBP_PROTEIN_LOCALIZATION_TO_CHROMOSOME_TELOMERIC_REGION	-1.5955	0.0161
GOBP_POSITIVE_REGULATION_OF_RESPONSE_TO_DNA_DAMAGE_STIMULUS	-1.5928	0.0165
GOBP_NUCLEOTIDE_EXCISION_REPAIR	-1.5897	0.0171
GOBP_REGULATION_OF_TELOMERE_MAINTENANCE_VIA_TELOMERASE	-1.5818	0.0187
GOBP_HISTONE_DEUBIQUITINATION	-1.5768	0.0198
GOMF_NUCLEASE_ACTIVITY	-1.5767	0.0198
GOBP_RNA_MODIFICATION	-1.5741	0.0203
GOBP_NEGATIVE_REGULATION_OF_ORGANELLE_ORGANIZATION	-1.5611	0.0234
GOBP_POSITIVE_REGULATION_OF_DOUBLE_STRAND_BREAK_REPAIR	-1.5481	0.0269
GOMF_LIGASE_ACTIVITY_FORMING_CARBON_OXYGEN_BONDS	-1.5315	0.032
GOBP_POSITIVE_REGULATION_OF_CELL_CYCLE_G1_S_PHASE_TRANSITION	-1.5225	0.035
GOBP_RESPONSE_TO_UV	-1.5026	0.0419
GOBP_TRNA_MODIFICATION	-1.4982	0.0432

Immune responses & regulation

EnrichmentMap::Name	EnrichmentMap::NES (Dataset 1)	EnrichmentMap::fdr_qvalue (Dataset 1)
GOBP_MYELOID_LEUKOCYTE_MEDIATED_IMMUNITY	2.6695	0
GOBP_NEGATIVE_REGULATION_OF_IMMUNE_EFFECTOR_PROCESS	2.6426	0
GOBP_NEGATIVE_REGULATION_OF_LEUKOCYTE_MEDIATED_IMMUNITY	2.6137	0
GOBP_NEGATIVE_REGULATION_OF_LYMPHOCYTE_MEDIATED_IMMUNITY	2.5713	0
GOBP_NEGATIVE_REGULATION_OF_LEUKOCYTE_PROLIFERATION	2.5329	0.0001
GOBP NEGATIVE REGULATION OF PRODUCTION OF MOLECULAR MEDIAT	2.523	0.0001
GOBP_NEGATIVE_REGULATION_OF_ADAPTIVE_IMMUNE_RESPONSE	2.4771	0.0001
GOBP_REGULATION_OF_T_HELPER_17_TYPE_IMMUNE_RESPONSE	2.4692	0.0002
GOBP_T_CELL_ACTIVATION_INVOLVED_IN_IMMUNE_RESPONSE	2.4576	0.0002
GOBP_NEGATIVE_REGULATION_OF_T_CELL_PROLIFERATION	2.4463	0.0002
GOBP_NEGATIVE_REGULATION_OF_CELL_ADHESION	2.3934	0.0003
GOBP_POSITIVE_REGULATION_OF_CELL_KILLING	2.3843	0.0002
GOBP_NEGATIVE_REGULATION_OF_CYTOKINE_PRODUCTION	2.3815	0.0003
GOBP_T_HELPER_17_TYPE_IMMUNE_RESPONSE	2.3739	0.0004
GOBP_ALPHA_BETA_T_CELL_PROLIFERATION	2.2916	0.0009
GOBP_NEGATIVE_REGULATION_OF_CELL_CELL_ADHESION	2.2802	0.0009
GOBP NEGATIVE REGULATION OF CYTOKINE PRODUCTION INVOLVED IN	2.2585	0.0013
GOBP POSITIVE REGULATION OF MYELOID LEUKOCYTE MEDIATED IMMU	2.2379	0.0014
GOBP_MACROPHAGE_CYTOKINE_PRODUCTION	2.2299	0.0015
GOBP_CYTOKINE_PRODUCTION_INVOLVED_IN_IMMUNE_RESPONSE	2.2163	0.0017
GOBP_REGULATION_OF_CELL_KILLING	2.2054	0.002
GOBP_NEGATIVE_REGULATION_OF_CELL_ACTIVATION	2.2037	0.002
GOBP_REGULATION_OF_LEUKOCYTE_PROLIFERATION	2.1933	0.002
GOBP_T_CELL_DIFFERENTIATION_INVOLVED_IN_IMMUNE_RESPONSE	2.1821	0.0023
GOBP_NEGATIVE_REGULATION_OF_LEUKOCYTE_CELL_CELL_ADHESION	2.16	0.0027
GOBP_ALPHA_BETA_T_CELL_ACTIVATION	2.1556	0.0027
GOBP_CD4_POSITIVE_ALPHA_BETA_T_CELL_ACTIVATION	2.1498	0.0028
GOBP_CELL_KILLING	2.1111	0.0038
GOBP_MYELOID_LEUKOCYTE_CYTOKINE_PRODUCTION	2.1004	0.004
GOBP_ACTIVATED_T_CELL_PROLIFERATION	2.0907	0.0043
GOBP_T_HELPER_17_CELL_DIFFERENTIATION	2.0793	0.0046
GOBP_REGULATION_OF_T_CELL_CYTOKINE_PRODUCTION	2.0791	0.0046
GOBP_REGULATION_OF_T_CELL_PROLIFERATION	2.0698	0.0049
GOBP_T_CELL_CYTOKINE_PRODUCTION	2.062	0.0052
GOBP_POSITIVE_REGULATION_OF_ALPHA_BETA_T_CELL_DIFFERENTIATION	2.0548	0.0055
GOBP_TOLERANCE_INDUCTION	2.0436	0.0059
GOBP_POSITIVE_REGULATION_OF_DEFENSE_RESPONSE	2.0357	0.006
GOBP_REGULATION_OF_INNATE_IMMUNE_RESPONSE	2.0278	0.0064
GOBP POSITIVE REGULATION OF CD4 POSITIVE ALPHA BETA T CELL ACT	2.0235	0.0064
GOBP_T_CELL_PROLIFERATION	2.0137	0.0065
GOBP POSITIVE REGULATION OF CD4 POSITIVE ALPHA BETA T CELL DIF	2.0085	0.0065
GOBP_REGULATION_OF_IMMUNE_EFFECTOR_PROCESS	1.9823	0.0074
GOBP ADAPTIVE IMMUNE RESPONSE BASED ON SOMATIC RECOMBINAT	1.9664	0.0081
GOBP_POSITIVE_REGULATION_OF_CELL_ADHESION	1.9628	0.0084
GOBP_POSITIVE_REGULATION_OF_CELL_ACTIVATION	1.9587	0.0086
GOBP_NEGATIVE_REGULATION_OF_IMMUNE_RESPONSE	1.9527	0.0088
GOBP_NEGATIVE_REGULATION_OF_LYMPHOCYTE_ACTIVATION	1.9455	0.0091
GOBP_CD8_POSITIVE_ALPHA_BETA_T_CELL_ACTIVATION	1.9439	0.0091
GOBP_REGULATION_OF_ALPHA_BETA_T_CELL_ACTIVATION	1.9329	0.0098
GOBP_POSITIVE_REGULATION_OF_ALPHA_BETA_T_CELL_ACTIVATION	1.9313	0.0098
GOBP_TYPE_2_IMMUNE_RESPONSE	1.9161	0.0106
GOBP_NEGATIVE_REGULATION_OF_ALPHA_BETA_T_CELL_ACTIVATION	1.9152	0.0106
GOBP_POSITIVE_REGULATION_OF_HEMOPOIESIS	1.9117	0.0109
GOBP_ADAPTIVE_IMMUNE_RESPONSE	1.9026	0.0114
GOBP_LYMPHOCYTE_MEDIATED_IMMUNITY	1.8966	0.0119
GOBP_POSITIVE_REGULATION_OF_IMMUNE_EFFECTOR_PROCESS	1.8924	0.0121
GOBP_NEGATIVE_REGULATION_OF_IMMUNE_SYSTEM_PROCESS	1.8726	0.0133
GOBP_POSITIVE_REGULATION_OF_RESPONSE_TO_CYTOKINE_STIMULUS	1.8694	0.0136
GOBP_REGULATION_OF_CD4_POSITIVE_ALPHA_BETA_T_CELL_ACTIVATION	1.8594	0.0144
GOBP_LEUKOCYTE_MEDIATED_CYTOTOXICITY	1.8331	0.017
GOBP_T_CELL_MEDIATED_IMMUNITY	1.8035	0.0201
GOBP_POSITIVE_REGULATION_OF_IMMUNE_RESPONSE	1.7948	0.021
GOBP_REGULATION_OF_ADAPTIVE_IMMUNE_RESPONSE	1.79	0.0218
GOBP_REGULATION_OF_ACTIVATED_T_CELL_PROLIFERATION	1.766	0.0248
GOBP_REGULATION_OF_T_CELL_MEDIATED_IMMUNITY	1.7593	0.0255
GOBP_REGULATION_OF_LEUKOCYTE_MEDIATED_IMMUNITY	1.758	0.0257
GOBP_POSITIVE_REGULATION_OF_LEUKOCYTE_CELL_CELL_ADHESION	1.7511	0.0266
GOBP_REGULATION_OF_T_CELL_ACTIVATION	1.7464	0.0275
GOBP_REGULATION_OF_NATURAL_KILLER_CELL_MEDIATED_IMMUNITY	1.7441	0.028
GOBP_POSITIVE_REGULATION_OF_CELL_CELL_ADHESION	1.7244	0.0313
GOBP_HUMORAL_IMMUNE_RESPONSE	1.7103	0.0335
GOBP_REGULATION_OF_LYMPHOCYTE_MEDIATED_IMMUNITY	1.6625	0.0428
GOBP_POSITIVE_REGULATION_OF_RESPONSE_TO_BIOTIC_STIMULUS	1.6617	0.0429
GOBP_ALPHA_BETA_T_CELL_DIFFERENTIATION	1.6348	0.0484
GOBP_REGULATION_OF_ISOTYPE_SWITCHING	-1.6844	0.0055
GOBP IMMUNOGLOBULIN PRODUCTION INVOLVED IN IMMUNOGLOBULIN	-1.7371	0.0025
GOBP_POSITIVE_REGULATION_OF_ISOTYPE_SWITCHING	-1.7658	0.0016
GOBP_SOMATIC_DIVERSIFICATION_OF_IMMUNOGLOBULINS	-1.7965	0.001
GOBP SOMATIC DIVERSIFICATION OF IMMUNOGLOBULINS INVOLVED IN	-1.8103	0.0008
GOBP_SOMATIC_DIVERSIFICATION_OF_IMMUNE_RECEPTORS	-1.8636	0.0003

Cytokine_Chemokine production

EnrichmentMap::Name	EnrichmentMap::NES (Dataset 1)	EnrichmentMap::fdr_qvalue (Dataset 1)
GOBP_TUMOR_NECROSIS_FACTOR_SUPERFAMILY_CYTOKINE_PRODUCTION	2.8605	0
GOBP_POSITIVE_REGULATION_OF_TUMOR_NECROSIS_FACTOR_SUPERFAMILY_CYTOKINE_PRODUCTION	2.8155	0
GOBP_NEGATIVE_REGULATION_OF_INTERLEUKIN_6_PRODUCTION	2.6995	0
GOBP_POSITIVE_REGULATION_OF_CHEMOKINE_PRODUCTION	2.6772	0
GOBP_INTERLEUKIN_6_PRODUCTION	2.6089	0
GOBP_INTERLEUKIN_10_PRODUCTION	2.4208	0.0001
GOBP_POSITIVE_REGULATION_OF_INTERLEUKIN_6_PRODUCTION	2.3704	0.0004
GOBP_CHEMOKINE_PRODUCTION	2.3257	0.0006
GOBP_INTERLEUKIN_8_PRODUCTION	2.0957	0.0042
GOBP_POSITIVE_REGULATION_OF_INTERLEUKIN_8_PRODUCTION	1.8328	0.017

Supplemental Table S2. List of annotated genes involved in epigentics

A1cf	Cdyl2	Fbrsl1	Kdm5c	Parp1	Rnf8	Taf6l	Znhit1
Abraxas1	Cecr2	Foxa1	Kdm5d	Parp2	Rps6ka3	Taf7	Zranb3
Abraxas2	Cenpc1	Foxo1	Kdm6a	Parp3	Rps6ka4	Taf8	Zzz3
Actb	Chaf1a	Foxp1	Kdm6b	Paxip1	Rps6ka5	Taf9	
Actl6a	Chaf1b	Foxp2	Kdm7a	Pbk	Rrp8	Taf9b	
Actl6b	Chd1	Foxp3	Kdm8	Pbrm1	Rsf1	Tbl1xr1	
Actr3b	Chd1l	Foxp4	Keap1	Pcgf1	Ruvbl1	Tdg	
Actr5	Chd2	Gadd45a	Kmt2a	Pcgf2	Ruvbl2	Tdrd3	
Actr6	Chd3	Gadd45b	Kmt2b	Pcgf3	Rybp	Tdrd7	
Actr8	Chd4	Gadd45g	Kmt2c	Pcgf5	Safb	Tdrkh	
Adnp	Chd5	Gatad1	Kmt2d	Pcgf6	Sap130	Tet1	
Aebp2	Chd6	Gatad2a	Kmt2e	Pcna	Sap18	Tet2	
Aicda	Chd7	Gatad2b	Kmt5a	Pdp1	Sap25	Tet3	
Aire	Chd8	Gfi1	Kmt5b	Pelp1	Sap30	Tex10	
Alkbh1	Chd9	Gfi1b	Kmt5c	Phc1	Sap30l	Tfdp1	
Anp32a	Chek1	Glyr1	L3mbtl1	Phc2	Satb1	Tfpt	
Anp32b	Chrac1	Gse1	L3mbtl2	Phc3	Satb2	Tle1	
Anp32e	Chtop	Gtf2i	L3mbtl3	Phf1	Scmh1	Tle2	
Apbb1	Chuk	Gtf3c4	L3mbtl4	Phf10	Scml2	Tle4	
Apex1	Cir1	Haspin	Las1l	Phf12	Scml4	Tlk1	
Apobec1	Cit	Hat1	Lbr	Phf13	Senp1	Tlk2	
Apobec2	Clns1a	Hcfc1	Leo1	Phf14	Senp3	Tnp1	
Arid1a	Clock	Hcfc2	Lrwd1	Phf19	Set	Tnp2	
Arid1b	Crb2	Hdac1	Map3k7	Phf2	Setd1a	Tonsl	
Arid2	Crebbp	Hdac10	Mapkapk3	Phf20	Setd1b	Top2a	
Arid4a	Csnk2a1	Hdac11	Mastl	Phf20l1	Setd2	Top2b	
Arid4b	Ctbp1	Hdac2	Max	Phf21a	Setd3	Trim16	
Arntl	Ctbp2	Hdac3	Maz	Phf8	Setd5	Trim24	
Arrb1	Ctcf	Hdac4	Mbd1	Phip	Setd6	Trim27	
Asf1a	Ctcfl	Hdac5	Mbd2	Piwil4	Setd7	Trim28	
Asf1b	Ctr9	Hdac6	Mbd3	Pkm	Setdb1	Trim33	
Ash1l	Cul1	Hdac7	Mbd4	Pkn1	Setdb2	Trp53	
Ash2l	Cul2	Hdac8	Mbd5	Pogz	Setmar	Trp53bp1	
Asxl1	Cul3	Hdac9	Mbd6	Pole3	Sf3b1	Trrap	
Asxl2	Cul4a	Hdgf	Mbip	Ppargc1a	Sf3b3	Tssk6	
Asxl3	Cul4b	Hdgfl2	Mbtd1	Ppm1g	Sfmbt1	Ttk	
Atad2	Cul5	Hells	Mcrs1	Ppp2ca	Sfmbt2	Tyw5	
Atad2b	Cxxc1	Hif1an	Mdc1	Ppp4c	Sfpq	Ube2a	
Atf2	Dapk3	Hinfp	Meaf6	Ppp4r2	Sgf29	Ube2b	
Atf7ip	Daxx	Hira	Mecp2	Ppp4r3a	Shprh	Ube2d1	
Atm	Ddb1	Hirip3	Men1	Ppp4r3b	Sin3a	Ube2d3	
Atn1	Ddb2	Hjurp	Mga	Prdm1	Sin3b	Ube2e1	
Atr	Ddx21	Hlcs	Mllt1	Prdm11	Sirt1	Ube2h	
Atrx	Ddx50	Hltf	Mllt10	Prdm12	Sirt2	Ube2n	
Atxn7	Dek	Hmg20a	Mllt6	Prdm13	Sirt6	Ube2t	
Atxn7l3	Dmap1	Hmg20b	Morf4l1	Prdm14	Sirt7	Ubn1	

Aurka	Dnajc1	Hmgb1	Morf4l2	Prdm16	Skp1	Ubr2
Aurkb	Dnajc2	Hmgn1	Mov10	Prdm2	Slf1	Ubr5
Aurkc	Dnd1	Hmgn2	Mphosph8	Prdm4	Smarca1	Ubr7
Babam1	Dnmt1	Hmgn2-ps1	Mrgbp	Prdm5	Smarca2	Uchl5
Babam2	Dnmt3a	Hmgn3	Msh6	Prdm6	Smarca4	Uhrf1
Bahd1	Dnmt3b	Hmgn5	Msl1	Prdm8	Smarca5	Uhrf2
Banp	Dnmt3l	Hp1bp3	Msl2	Prdm9	Smarcad1	Uimc1
Bap1	Dnttip2	Hr	Msl3	Prkaa1	Smarcal1	Usp11
Bard1	Dot1l	Hspa1a	Mst1	Prkaa2	Smarcb1	Usp12
Baz1a	Dpf1	Hspa1b	Mta1	Prkab1	Smarcc1	Usp15
Baz1b	Dpf2	Huwe1	Mta2	Prkab2	Smarcc2	Usp16
Baz2a	Dpf3	Ikzf1	Mta3	Prkag1	Smarcd1	Usp17le
Baz2b	Dppa3	Ikzf3	Mtf2	Prkag2	Smarcd2	Usp21
Bcor	Dpy30	Ing1	Mybbp1a	Prkag3	Smarcd3	Usp22
Bcorl1	Dr1	Ing2	Myo1c	Prkca	Smarce1	Usp3
Bmi1	Dtx3l	Ing3	Mysm1	Prkcb	Smyd1	Usp36
Bptf	Dzip3	Ing4	Naa60	Prkcd	Smyd2	Usp44
Brca1	E2f6	Ing5	Nap1l1	Prkdc	Smyd3	Usp46
Brca2	Eed	Ino80	Nap1l2	Prmt1	Smyd4	Usp49
Brcc3	Ehmt1	Ino80b	Nap1l4	Prmt2	Snai2	Usp7
Brd1	Ehmt2	Ino80c	Nasp	Prmt5	Sp1	Uty
Brd2	Eid1	Ino80d	Nat10	Prmt6	Sp100	Vdr
Brd3	Eid2	Ino80e	Nbn	Prmt7	Sp140	Vps72
Brd4	Eid2b	Jade1	Ncl	Prmt8	Spen	Vrk1
Brd7	Elp1	Jade2	Ncoa1	Prmt9	Spop	Wac
Brd8	Elp2	Jade3	Ncoa2	Prpf31	Srcap	Wdr5
Brd9	Elp3	Jak2	Ncoa3	Prr14	Srsf1	Wdr77
Brdt	Elp4	Jarid2	Ncoa6	Psip1	Srsf3	Wdr82
Brms1	Elp5	Jdp2	Ncor1	Pwwp3a	Ss18l1	Wsb2
Brms1l	Elp6	Jmjd1c	Ncor2	Rad51	Ss18l2	Yaf2
Brpf1	Eny2	Jmjd6	Nek6	Rad54b	Ssrp1	Yeats2
Brpf3	Ep300	Kansl1	Nek9	Rad54l	Stk4	Yeats4
Brwd1	Ep400	Kansl2	Nfrkb	Rad54l2	Suds3	Ywhab
Brwd3	Epc1	Kansl3	Nfyb	Rag1	Supt16	Ywhae
Bub1	Epc2	Kat14	Nfyc	Rag2	Supt3	Ywhaz
Carm1	Erbb4	Kat2a	Nipbl	Rai1	Supt6	Yy1
Cbx1	Ercc6	Kat2b	Noc2l	Rara	Supt7l	Zbtb16
Cbx2	Exosc1	Kat5	Npas2	Rb1	Suv39h1	Zbtb33
Cbx3	Exosc2	Kat6a	Npm1	Rbbp4	Suv39h2	Zbtb7c
Cbx4	Exosc3	Kat6b	Npm2	Rbbp5	Suz12	Zcwpw1
Cbx5	Exosc4	Kat7	Nsd1	Rbbp7	Syncrip	Zfp217
Cbx6	Exosc5	Kat8	Nsd2	Rbx1	Tada1	Zfp516
Cbx7	Exosc6	Kdm1a	Nsd3	Rcc1	Tada2a	Zfp532
Cbx8	Exosc7	Kdm1b	Nsl1	Rcor1	Tada2b	Zfp541
Cdc6	Exosc8	Kdm2a	Oga	Rcor3	Tada3	Zfp57
Cdc73	Exosc9	Kdm2b	Ogt	Rest	Taf1	Zfp592
Cdk1	Eya1	Kdm3a	Padi1	Ring1	Taf10	Zfp687

Cdk17	Eya2	Kdm3b	Padi2	Riox2	Taf12	Zfp711
Cdk2	Eya3	Kdm4a	Padi3	Rlim	Taf2	Zgpat
Cdk3	Eya4	Kdm4b	Padi4	Rmi1	Taf3	Zhx1
Cdk5	Ezh1	Kdm4c	Paf1	Rnf168	Taf4	Zmym2
Cdk7	Ezh2	Kdm4d	Pagr1a	Rnf2	Taf5	Zmym3
Cdk9	Fbl	Kdm5a	Pak2	Rnf20	Taf5l	Zmynd11
Cdyl	Fbrs	Kdm5b	Parg	Rnf40	Taf6	Zmynd8

Supplemental Table S3. Read counts of 30 genes significantly changed by U0126.

SYMBOL	C1	C2	C3	U1	U2	U3	Comments
Prmt2	504.0	403.2	505.6	899.3	836.0	1274.6	Histone methylation
Phf19	3197.4	3405.9	3848.6	1865.2	1605.7	1003.8	H3K27
Ezh2	12041.6	12168.1	12337.1	6991.4	7089.5	3194.9	H3K27
Cbx5	30434.4	31377.1	33654.8	17077.6	16664.0	11174.5	H3K9
Suv39h1	7808.9	7542.1	7659.0	4092.7	4142.0	2263.3	H3K9
Kdm7a	8682.9	9133.1	11342.1	18807.4	18931.1	23798.9	H3K9
Kdm5b	251.1	258.4	322.3	1216.5	1309.4	1975.2	H3K4
Bard1	2296.3	2267.1	2651.7	1391.2	1304.0	678.7	Bst Ca
Brca2	5036.7	5463.6	5608.7	2986.7	2741.9	1693.7	Bst Ca
Atad2	22155.0	22609.8	19975.8	11831.4	12081.0	5094.0	ATPases
Aurka	12364.3	12738.0	17338.5	7502.3	7621.1	4128.6	ATPases
Chek1	3780.3	3931.0	3381.2	2105.2	1997.9	812.6	ATPases
Cit	3256.3	3398.6	4740.1	2105.2	2115.6	1103.1	ATPases
Mastl	1731.5	1885.8	2408.2	1234.3	1094.3	680.2	ATPases
Aicda	29.0	9.4	35.6	148.5	71.7	212.2	Nucleotide modifiers
Apobec1	12148.6	12356.7	13395.8	41273.8	40800.3	49749.8	Nucleotide modifiers
Rad51	6928.7	6821.2	6138.9	3600.9	3431.8	1383.0	DNA repair
Rad54b	2128.6	1893.1	1581.2	916.0	933.4	435.7	DNA repair
Rad54l	5380.3	5617.8	5252.4	3055.6	2832.6	1427.4	DNA repair
Gadd45a	66.2	52.1	30.5	123.6	132.6	91.8	DNA repair
Parp3	674.5	657.4	754.1	2132.5	2256.3	3123.4	DNA repair
Jade1	6691.2	6733.6	7543.6	3547.4	3335.8	2583.1	Transcription coactivator
Eya4	185.8	160.4	263.8	873.2	934.7	1008.3	Transcription coactivator
Hirip3	17489.0	16808.6	15043.9	8945.7	8159.5	4872.1	Transcription coactivator
Hmgb1	25996.0	25996.9	25248.6	14490.2	14471.2	8364.1	Transcription coactivator
Hmgn5	4873.6	4621.8	5833.5	2806.1	2395.6	2099.3	Transcription coactivator
Nasp	27771.0	27681.6	23125.4	14714.7	14498.3	6700.5	Transcription coactivator
Prdm1	14.5	9.4	20.4	165.1	147.4	169.3	Repressor
Ube2t	1436.9	1316.9	1979.8	872.0	925.2	460.5	Ubiquitin modifier
Usp44	3.6	6.3	11.9	1.2	0.0	0.0	Ubiquitin modifier

Supplemental Table S4: List of qPCR Primers

Primers	Sequences
mGAPDH	F: GCATTGTGGAAGGGCTCATG<br>R: TTGCTGTTGAAGTCGCAGGAG
mB2M	F: ACCGTCTACTGGGATCGAGA<br>R: TGCTATTTCTTTCTGCGTGCAT"
mIL-1β	F: GTGGACCTTCCAGGATGAGG<br>R: GCTTGGGATCCACACTCTCC
mIL-6	F: GTGGAAATGAGAAAAGAGTTGTGC<br>R: CCAGGTAGCTATGGTACTCCAG
mTNF	F: ATGAGAAGTTCCCAAATGGCC<br>R: TCCACTTGGTGGTTTGCTACG
mCXCL10	F: TGATGGTCAAGCCATGGTCC<br>R: GTCGCACCTCCACATAGCTT
mCBX5	F: ATCACCACTGCCTGGCTAAC<br>R: AACCAAGGCTGGACAAACCA
mSUV39H1	F: AGATGCCAGGTCTGTGTGTG<br>R: GGCTGAGGGGTACAGAGAGA
mKDM7a	F: TTTGGTGGGACTTCAGTCTGG<br>R: GCCAAATTTTCATTTGTTGGCT

List of ChIP-qPCR Primers

Primers	Sequences	Target Region
mIL-1β H3K9me3 “Upstream of TSS Site” primer	F: TCAAGATGGTCGTCTGTTCG<br>R: GGATGACTCTGCCAAAAAGG	chr2:129204402-129205100
mIL-1β H3K9me3 “Intragenic Site” primer	F: TGACGCCATGTGGCATTAGA<br>R: AAGAGCCATGTTGCATCCCA	chr2:129194684-129194925
mIL-6 H3K9me3 “Upstream of TSS Site” primer	F: CCAATGGAGGAGCTAGAGAAAG<br>R: GTGGTGGTAACTGGTTAGTTCA	chr5:30331801-30332513
mIL-6 H3K9me3 “Intragenic Site” primer	F: GAGGGACATCTGGGTTCTTTC<br>R: GCAGATGCTGGTAAGGATGT	chr5:30342383-30343572
mTNF H3K9me3 “Upstream of TSS Site” primer	F: TGGATGGATCTCCCTAGCTCA<br>R: GTGGATTCACGGGAGTGAGG	chr17:35339892-35340550
mTNF H3K9me3 “Intragenic Site” primer	F: CCGGACTCCGCAAAGTCTAA<br>R: AGCCCATATACCTGGGAGGA	chr17:35336777-35337405
mCXCL10 H3K9me3 “Upstream of TSS Site” primer	F: CTCCAGAAGAGGGAGTCAGAT<br>R: TGGGCTTGGAATGTCGTAAG	chr5:92781622-92782198
mCXCL10 H3K9me3 “Intragenic Site” primer	F: GAAGGTTGGCTCGGGATGTC<br>R: GGACTCAGGGAGGGAAACTC	chr5:92777655-92778132
